# The Optimization of Operating Conditions in the Cross-Flow Microfiltration of Grape Marc Extract by Response Surface Methodology

**DOI:** 10.3390/foods13010020

**Published:** 2023-12-20

**Authors:** Karla Pérez, Alfredo Cassano, René Ruby-Figueroa

**Affiliations:** 1 Programa Institucional de Fomento a la Investigación, Desarrollo e Innovación, Universidad Tecnológica Metropolitana, Ignacio Valdivieso 2409, San Joaquín, Santiago 8940577, Chile; karla.perezr@utem.cl; 2 Institute on Membrane Technology, ITM-CNR, University of Calabria, via P. Bucci, 17/C, 87036 Rende, Italy

**Keywords:** microfiltration, grape marc, optimization, response surface methodology, clarification

## Abstract

The recovery of valuable compounds like phenolic compounds and sugars from grape marc extracts implies different steps, including clarification. In this study, a response surface methodology (RSM) was used as a statistical tool to study the effects of operating conditions such as transmembrane pressure (TMP), temperature and feed flow rate on the performance of a microfiltration (MF) monotubular ceramic membrane with a pore size of 0.14 μm in the clarification of grape marc extract from the Carménère variety, as well to optimize the process conditions by implementing the Box–Behnken statistical design. The desirability function approach was applied to analyze the regression model equations in order to maximize the permeate flux and concentration of malvidin-3-O-glucoside, glucose and fructose in the clarified extract. The optimal operating conditions were found to be 1 bar, 29.01 °C and 5.64 L/min. Under these conditions, the permeate flux and concentration of malvidin-3-O-glucoside, glucose and fructose resulted in 65.78 L/m^2^h, 43.73 mg/L, 305.89 mg/L, and 274.85 mg/L, respectively.

## 1. Introduction

The wine industry is a recognized agricultural activity that generates high amounts of organic wastes and by-products [1]. According to the annual report of the International Organization of Vine and Wine (OIV), the world’s vineyards produced a total of 80.1 Mton of fresh grapes in 2022, corresponding to a global wine production of 258 mhl [2].

Grape pomace is one of the most abundant solid by-products generated during winemaking: it is composed mainly of the skin and seeds of the fruit, representing approximately 20% of the mass of total processed grapes [3,4]. It has been estimated that the production of 6 L of wine generates 1 kg of grape pomace: this yield accounts for 10.5–13.1 Mton of grape pomace in the world annually [5]. These by-products must be properly disposed of because they can induce adverse effects on the environment due to their high levels of biochemical and chemical oxygen demand (BOD and COD) [6]. The current disposal procedures for grape pomace are composting [7,8] and the use as a feedstuff for ruminant animals [8]. The first approach is not economically viable due to a lack of some essential nutrients. On the other hand, the incorporation of grape pomace into the animal diet requires an evaluation of its effect on animal production by testing their outcomes on growth performance; in addition, grape pomace, being rich in proteins, cannot be digested by most animals and, therefore, used as a source of energy.

Grape pomace is considered to be of interest due to its high content of bioactive compounds such as phenolic compounds including flavonoids, stilbenes and tannins [9,10], as well as pectins [11] and sugars such as fructose, glucose and sucrose [8,12]. Phenolic compounds are secondary plant metabolites whose consumption has been correlated to the reduction of coronary heart disease and hypertension [13,14], improvement of blood pressure and glycemia [15] and lowering of cholesterol levels [16,17]. They present antiviral, antimicrobial and anti-inflammatory properties which vary with the variety of grapes [18] and can be used in food preservation without altering its sensory properties if applied in low concentrations [19,20].

Phenolic compounds do not suffer significant chemical alterations during wine making and about 70% of phenolic compounds present in the grape remain in the pomace [21]. Therefore, the recovery of these compounds from grape by-products offers a great opportunity to promote the development of value-added products (such as food additives, nutraceuticals, dietary supplements, antimicrobial components and cosmetics) providing alternatives to minimize the environmental impact of wine-making activities as well as an economic gain from the revaluation of by-products [22].

Several investigations have been reported in the literature focusing on innovative methodologies to valorize grape pomace and, more generally, winery by-products. At the beginning of 2020, the Viña Concha y Toro, located in Chile, signed an agreement to deliver the grape pomace obtained from its wine-making processes for the subsequent production of flour [23]. Rivera et al. [24] proposed the extraction of monomeric sugars from the distillation of grape pomace for the production of lactic acid (as a medium for its fermentation) and biosurfactants. Sandoval et al. [25] proposed the use of grape pomace from Cabernet Sauvignon to reduce the asphalt pavement damage. Dehydrated and powdered grape pomace act as an antioxidant and as filler component, particularly above 10% addition.

Microwave-assisted extraction (MAE) [26,27,28], ultrasound-assisted extraction (UAE) [29,30,31] and supercritical fluid extraction (SFE) [32,33,34] are emerging technologies for extracting bioactive compounds, including phenolic compounds, from plant matrices. These “green” techniques are able to minimize the limitations arising from conventional techniques (i.e., maceration, Soxhlet extraction and hydrodistillation) such as the formation of toxic residues, long extraction times and high solvent and energy consumption [22,31].

Extraction procedures result in obtaining mixtures of different compounds; therefore, purification methods are needed for the isolation of target compounds without affecting their structure and function, which ultimately translates into their bioactivity. Pressure-driven membrane operations represent useful approaches in this regard because they can operate under mild conditions of temperature and pressure, without involving the addition of chemicals or solvents to the feed stream, avoiding product contamination and preserving the biological activity of the compounds of interest. These processes are based on a sieving mechanism involving the physical separation of analytes based on particle or molecular size [35]. Consequently, they do not require a phase change or high temperatures to reach high selectivity [36]. Therefore, they offer significant advantages over conventional processes such as distillation, ion exchange, precipitation, coagulation/flocculation and adsorption, especially in terms of low energy consumption, a reduced number of processing steps, greater separation efficiency, and improved end-product quality.

Pressure-driven membrane operations have been demonstrated to be a useful approach for the recovery of phenolic-based molecules from agro-food residues, delivering high yields in terms of flux and the retention of bioactive compounds [37,38,39]. In particular, microfiltration (MF) and ultrafiltration (UF) processes are useful approaches to remove suspended solids and colloids from the original stream or raw extracts; UF and nanofiltration (NF) can be used to selectively separate phenolic compounds from other compounds (e.g., sugars, proteins, lipids); NF and reverse osmosis (RO) can be used to concentrate polyphenol-rich streams [35]. The performance of these processes in terms of productivity and selectivity towards phenolic compounds is affected by several parameters including operating and fluid-dynamic conditions (i.e., transmembrane pressure (TMP), feed flow rate and temperature) as well as membrane materials and properties (hydrophilicity/hydrophobicity, surface topography, charge and pore size) which have a strong influence on membrane–solute interactions, and hence on membrane fouling phenomena [37,40,41]. Therefore, the optimization of these parameters is crucial to improve their efficiency. In this regard, applying statistical techniques such as the response surface methodology (RSM) can be helpful in determining the effects of the operational variables and the search for their optimal levels [42]. This approach allows for the estimation of the response surface that approximates the relationship between the independent and response variables of the process, generally fitting a second-degree model using Ordinary Least Squares (OLS). The RSM has already been applied to improve extraction and purification processes, such as the recovery of phenolic compounds by UAE from residues from the olive oil industry [42] and from pomegranate peels [43], the removal of contaminants in industrial wastewater by means of forward osmosis and UF membranes [44] and the concentration of saponins by separation using ultrasound-assisted UF membranes [45], among others.

In view of the foregoing, the current study aimed at evaluating both single and interaction effects of operating conditions such as transmembrane pressure, temperature and feed flow rate on the permeate flux and recovery of sugars and phenolic compounds in the microfiltration of grape marc by using the RSM approach. In a previous work [12], malvidin-3-O-glucoside was found to be one of the most representative anthocyanin of the grape marc extract from red grape Carménère. This compound, together with malvidin 3-(coumaroyl)-glucoside and malvidin 3-(acetyl)-glucoside, contributes to the distinctive characteristics of Carménère grapes regarding astringency and color [46]. In light of this information, this compound was chosen as the reference compound to evaluate the effect of operating parameters on the recovery of phenolic compounds in the clarified extract.

## 2. Materials and Methods

### 2.1. Grape Marc Extract

Grape marc from red grape (Carménère variety) was kindly provided by Concha y Toro wine maker (Pencahue, Chile) and stored at −80 °C before use. The extraction process was carried out by ultrasound-assisted extraction (UAE) under optimal operating conditions (grape marc, 23.85% (*w*/*w*); ethanol, 40% (*w*/*w*); water, 36.15% (*w*/*w*); amplitude, 20%; temperature, 22 °C; operating time, 15 min) identified in a previous work [12]. The extract was filtered through nylon cloth and stored at −5 °C until its clarification by MF.

### 2.2. MF Set-Up and Procedures

**Figure 1:**
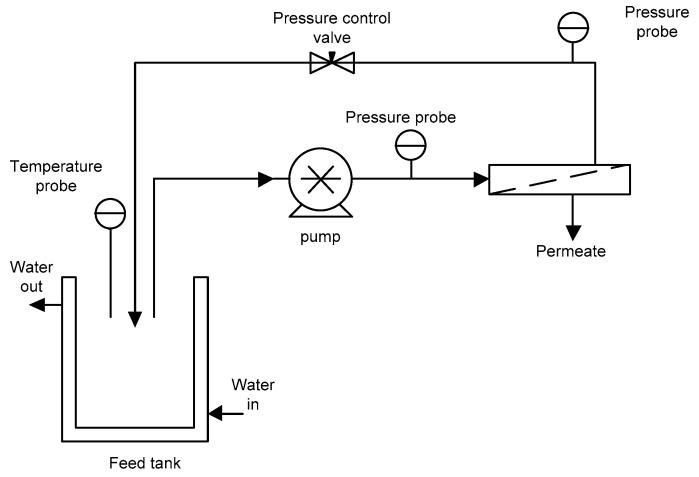
Schematic diagram of MF experimental set-up.

The MF experiments were carried out using a laboratory unit (Figure 1) equipped with a mono-tubular ceramic membrane (Tami Industries, Nyons, France) with a pore size of 0.14 µm and an effective membrane area of 0.005 m^2^. The temperature of the extract was controlled by circulating tap water in a two-layered feed tank.

The MF system was operated according to the Box–Behnken experimental design in a batch concentration configuration in which the permeate is collected separately while the retentate is continuously recycled back to the feed tank. For each filtration experiment, lasting 180 min, the permeate flux, expressed as L/m^2^h, was continuously monitored by measuring the permeate volume collected in a certain time according to the following equation:

(1) $$J=\frac{V}{At}$$

where *A* is the membrane surface area (m^2^), *V* is the collected permeate volume (L) and *t* (h) is the operating time.

### 2.3. Analytical Measurements

#### 2.3.1. Anthocyanins Analysis

The identification of anthocyanins in the UAE extract was performed by HPLC-DAD-ESI-MS/MS, as reported elsewhere [12]. The separation was carried out by using a Waters ACCQ-TAGTM ULTRA C18 column (2.1 × 100 mm, 1.7 µm) and the chromatographic conditions were established according to Ruiz et al. [47], with slight modifications. The detection wavelength was 520 nm and a malvidin-3-glucoside external calibration curve was used for quantification. Results were expressed as malvidin-3-O-glucoside equivalents.

#### 2.3.2. Glucose and Fructose Quantification

The quantitative determination of glucose and fructose was carried out by using a UPLC^®^ system (PerkinElmer Altus TM A-30 PerkinElmer Inc., Waltham, MA, USA) equipped with a Brownlee Analytical Amino column (4.6 mm × 150 mm, 5 µm) [48]. Samples were eluted using a mixture of acetonitrile/water (75:25). Test conditions were the following: flow rate 1 mL/min, injection volume 5 µL and temperature 40 °C, respectively. An external calibration curve was used for the quantification of glucose and fructose.

### 2.4. Experimental Design and Data Analysis

Operating variables were selected according to the capability of the experimental set-up within the following range: temperature 20–30 °C, feed flow rate 3.19–6.67 L/min and TMP 1.0–3.5 bar (Table 1). Each factor’s limit (minimum and maximum) selection was based on a series of restrictions. We avoided working at pressures higher than 3.5 bar (value around the critical transmembrane pressure) to minimize membrane fouling. On the other hand, the temperature range was selected to prevent the degradation of thermosensitive compounds (phenolic compounds). Experiments were performed by using a Box–Behnken design composed of 15 runs, including three central points, with 5 degrees of freedom for the error. The correlation of the operating variables and the responses were fitted to a quadratic polynomial equation using the least squares method [49]. Statistic computations were carried out by using the software Statgraphics Centurion 19.2.01 (Statgraphics Technologies, The Plains, VA, USA)

## 3. Results and Discussion

The effect of three different operating conditions, including TMP, temperature and feed flow rate on permeate flux and the concentration of malvidin-3-O-glucoside and sugars (glucose and fructose) in the clarified extract was studied. Table 2 shows the results obtained for all the experiments according to the Box–Behnken design.

The linear, interaction and quadratic effects of the investigated factors on the responses were obtained by using analyses of variance (ANOVA). It should be pointed out that the MF with a membrane of 0.14 µm produced a clarified extract with a turbidity lower than 11.26 NTU, corresponding to a reduction in turbidity in the raw extract higher than 98%.

### 3.1. The Effect of Operating Conditions on Permeate Flux

The obtained model describing the effect of operating variables on the permeate flux showed an R-squared value of 97.9% and the lack-of-fit test proved that the model is adequate for the observed data at the 95% confidence level (*p* = 0.3338). The standard error was equal to 3.94908. The *p*-value of the Durbin–Watson statistic test resulted in greater than 5.0% (*p* = 0.3338), highlighting no serial autocorrelation in the residuals at the 5.0% significance level. 

**Figure 2:**
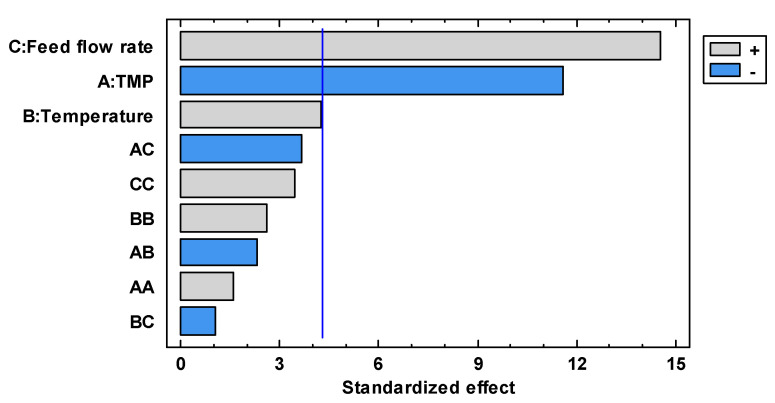
Standardized Pareto chart for the permeate flux.

Figure 2 shows the linear, quadratic and interaction effect of each studied factor plotted in the form of a Pareto chart: the effect is significant if its corresponding bar crosses the vertical line at the *p* = 0.05 level. The linear coefficients of feed flow rate were found to be the most critical effect in increasing the permeate flux, followed by the linear coefficient of TMP (whose increment produces a decrease in the permeate flux) and the linear effect of temperature (which produces an increase in the permeate flux). On the other hand, the interaction factors and quadratic effect did not produce a significant effect (*p* ≥ 0.05) on the permeate flux.

**Figure 3:**
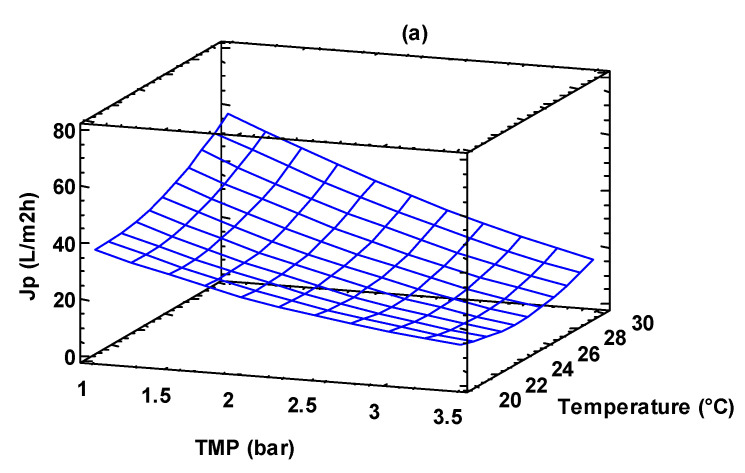

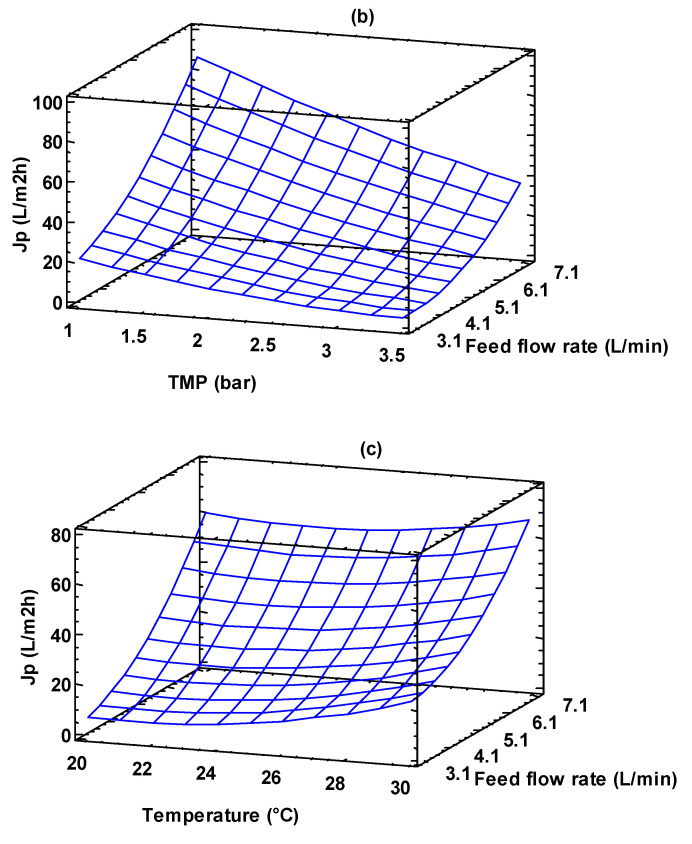
Response surface plot of permeate flux as a function of: (**a**) TMP and temperature; TMP and feed flow rate; (**b**) temperature and feed flow rate; (**c**) temperature and feed flow rate.

The quadratic regression equation describing the effect of the process variables on the permeate flux in terms of coded levels is expressed as:

(2) $$Y_{1}=59.8405+12.2687X_{1}-6.77493X_{2}+1.85948X_{3}+2.104X_{1}^{2}+0.2153X_{2}^{2}\;\;\;\;\;\;\;+2.34592X_{3}^{2}-0.7344X_{1}X_{2}-3.31494X_{1}X_{3}-0.233908X_{2}X_{3}$$

where *Y*_1_ is the predictive permeate flux for the MF process. Figure 3 shows the effect of the investigated variables on the permeate flux; the 3D plot, obtained using Equation (2), helps to predict the response at any combination of operating variables. The response surface of the permeate flux is plotted against two operating variables, while the third variable is kept constant (level 0 in Table 1).

The permeate flux decreased by increasing the TMP value for all the studied temperature and feed flow rate conditions. This phenomenon can be attributed to the formation of a viscous and gelatinous-type layer on the membrane surface, according to the gel polarization model, which creates an additional resistance to the permeate flux in addition to that of the membrane [50]. On the other hand, the enhanced shear stress at the membrane surface at higher feed flow rates increased the rate of the removal of deposited particles responsible of flux decay, so improving the membrane productivity [51]. An increase in the operating temperature led to an improvement in the permeation rate due to the reduction of the feed viscosity and the increase in the diffusion coefficient of macromolecules; however, that increase was not statistically significant.

### 3.2. The Effect of Operating Conditions on the Retention of Sugars

The model obtained for the glucose concentration in the permeate stream showed an R-squared value of 91.2% and the lack-of-fit test proved that the model is adequate for the observed data at the 95% confidence level (*p* = 0.7104). The standard error was equal to 5.59892, and there was no presence of autocorrelation in the residuals because the Durbin-Watson (DW) statistic showed a *p*-value higher than 0.05 (*p* = 0.0787). On the other hand, the model obtained for the fructose showed a better performance with an R-squared of 99.2%, and the lack-of-fit test proved that the model is adequate for the observed data at the 95% confidence level (*p* = 0.6767). The standard error was equal to 4.64384; the *p*-value of the Durbin–Watson statistic test resulted in greater than 5.0% (*p* = 0.1983), highlighting no serial autocorrelation in the residuals at the 5.0% significance level.

**Figure 4:**
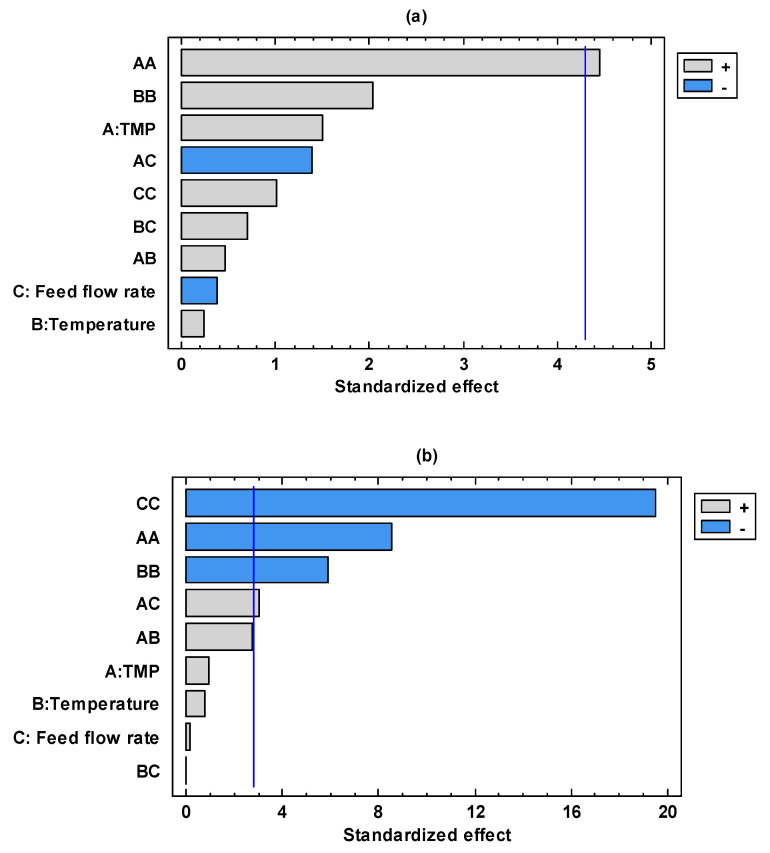
Standardized Pareto chart for: (**a**) glucose and (**b**) fructose concentration in the permeate stream.

Figure 4a,b shows the linear, quadratic, and interaction effect of each studied factor plotted in the form of a Pareto chart. In the case of glucose, the quadratic effect of TMP was found to be the significant factor (Figure 4a). On the other hand, Figure 4b shows that more factors were found to be significant for fructose. In this case, the quadratic effect of TMP, temperature, and feed flow rate showed a decrease in fructose concentration in the permeate stream as they increased. On the contrary, the interaction between TMP and feed flow rate showed an increase in the fructose concentration as it increased. These results can be explained assuming that even though glucose and fructose have the same molecular mass (about 180 g/mol), the differences in their structure result in distinct chemical and physical properties, including differences in isomerism and ring structure.

### 3.3. The Effect of Operating Conditions on Phenolic Compounds

The model obtained for the malvidin-3-O-glucoside concentration in the clarified extract showed an R-squared value of 52.35%; the lack-of-fit test proved that the model is adequate for the observed data at the 95% confidence level (*p* = 0.4528). The standard error was equal to 0.905373; the *p*-value of the Durbin–Watson statistic test resulted in greater than 5.0% (*p* = 0.7349), highlighting no serial autocorrelation in the residuals at the 5.0% significance level.

**Figure 5:**
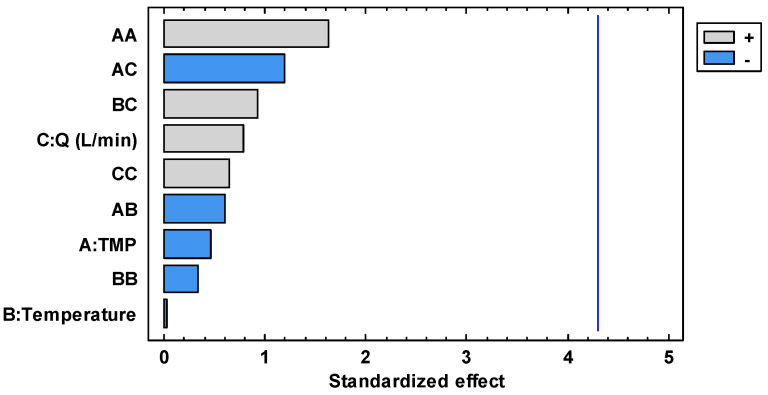
Standardized Pareto chart for malvidin-3-O-glucoside concentration in the permeate stream.

According to the Pareto chart illustrated in Figure 5, none of the studied factors has a statistically significant effect. However, the quadratic effect of TMP was the most important, followed by the interaction between TMP and feed flow rate.

The quadratic regression equation describing the effect of the process variables on the concentration of malvidin-3-O-glucoside in terms of coded levels is reported in the following:

(3) $$\begin{array}{c} Y_{2}=43.49-0.027467X_{1}+0.1797X_{2}-1.50383X_{3}+0.4904X_{1}^{2}-0.00645X_{2}^{2}\\ \;\;\;\;\;\;+0.101153X_{3}^{2}-0.0432X_{1}X_{2}-0.247126X_{1}X_{3}\\ \;\;\;\;\;\;+0.04827X_{2}X_{3} \end{array}$$

in which *Y*_2_ is the predictive concentration of malvidin-3-O-glucoside in the microfiltered extract.

**Figure 6:**
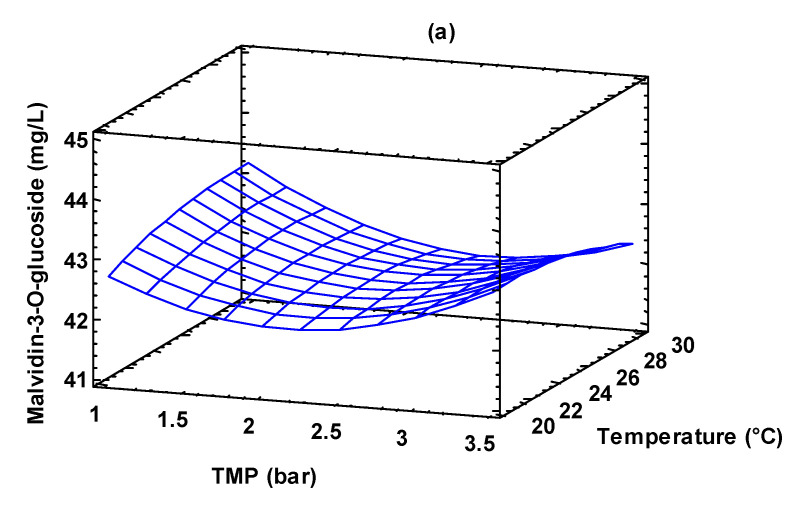

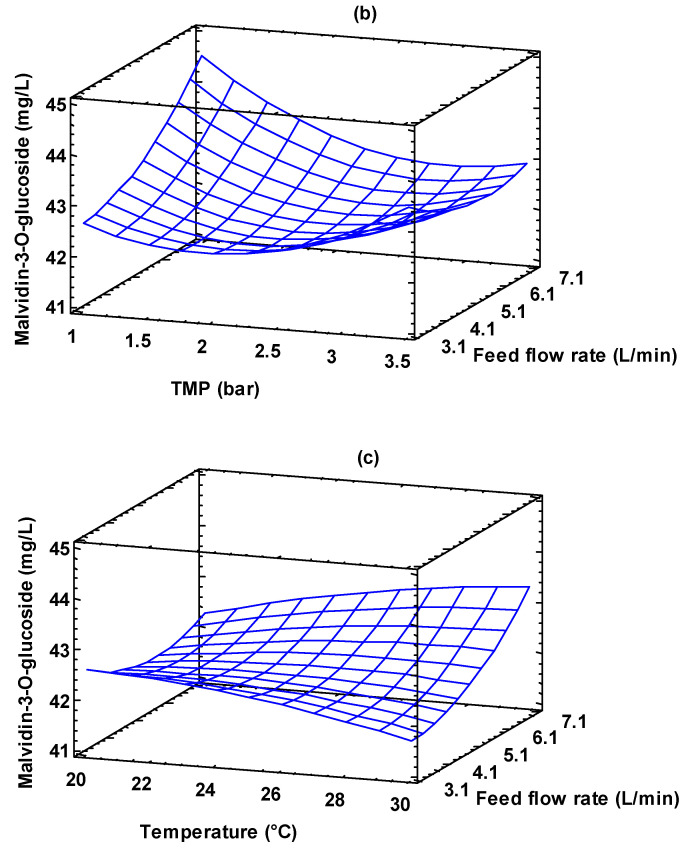
Response surface plot of malvidin-3-O-glucoside concentration as a function of: (**a**) TMP and temperature; TMP and feed flow rate; (**b**) temperature and feed flow rate; (**c**) temperature and feed flow rate.

The 3D plot obtained by using Equation (3) helps to predict the response at any combination of operating variables (Figure 6). In particular, the concentration of malvidin-3-O-glucoside decreased by increasing the TMP value up to 2.5 bar, while it increased for higher pressure values. This behavior can be attributed to the increased amount of deposited fouling materials on the membrane surface when the TMP was increased up to 2.5 bar and a higher amount of solute flowing through the membrane when the TMP was further raised. Similar trends were observed by Baklouti et al. [52] in the clarification of pomegranate juice with a ceramic UF membrane in a tubular configuration. On the other hand, temperature and feed flow rate did not show a significant effect on the malvidin-3-O-glucoside concentration (the impact of feed flow rate was more evident at 1 bar and 30 °C).

### 3.4. The Optimization of Multiple Responses

The multiple optimization was performed by using the desirability function (DF) in order to find the operating conditions that provide the “most desirable” responses. It is based on conducting experiments and fitting response models (yk) for all k responses, defining individual desirability functions for each response (dk) and maximizing the overall desirability compared to controllable factors. For each response yk(xi), a desirability function dk(yk) assigns numbers between 0 and 1 to the possible values of yk, with dk(yk) = 0 representing an utterly undesirable value of yk and dk(yk) = 1 representing a wholly desirable or ideal response value. In the present work, the minimum and maximum limits used for the permeate flux were 5.21 L/m^2^h (dk = 0) and 75.11 L/m^2^h (dk = 1), respectively. The limits used for the glucose concentration were 284.62 mg/L (dk = 0) and 316.47 mg/L (dk = 1), while for the fructose concentrations, limits were 244.79 mg/L (dk = 0) and 323.69 mg/L (dk = 1), respectively. In addition, the limits used for the concentration of malvidin-3-O-glucoside were 41.28 mg/L (dk = 0) and 44.21 mg/L (dk = 1), respectively. The results of the optimization, summarized in Table 3, highlight that under operating conditions of 1 bar, 29.01 °C and 5.64 L/min permeate flux and concentration of malvidin-3-O-glucoside, glucose and fructose were of 65.78 L/m^2^h, 43.73 mg/L, 305.89 mg/L and 274.85 mg/L, respectively.

## 4. Conclusions

The response surface methodology was used to study the effect of operating variables such as transmembrane pressure (TMP), temperature and feed flow rate on the performance of a 0.14 μm MF membrane in the clarification of grape marc extract. A Box–Behnken design was used for regression modeling and optimizing the MF operating variables. The optimization of multiple responses allowed us to establish the operating conditions, giving maximum membrane productivity (permeate flux) and concentration of malvidin-3-O-glucoside and sugars (glucose and fructose) in the permeate stream simultaneously. For an overall desirability of 0.66, a permeate flux of 65.78 L/m^2^h, concentration of malvidin-3-O-glucoside equal to 43.73 mg/L, concentration of glucose of 305.89 mg/L and concentration of fructose of 274.85 mg/L were estimated, respectively, in optimized operating variables (TMP = 1.0 bar, temperature = 29.01 °C and feed flow rate = 5.64 L/min).

The whole set of results provides the basis for the implementation of a green extraction and purification methodology of bioactive compounds from grape marc and, more generally, from wine by-products. Future research trends aim at the investigation of selected methodologies on a large scale as well to the combination of MF with other technologies (i.e., chromatographic separations or nanofiltration) to improve the purity of target compounds.

## Figures and Tables

**Table 1:** Experimental range and levels of the independent variables for Box–Behnken design.

		Variation Levels		
Parameters	Code	−1	0	1
TMP (bar)	X_1_	1.0	2.25	3.5
Temperature (°C)	X_2_	20	25	30
Feed flow rate (L/min)	X_3_	3.19	4.93	6.67

**Table 2:** Experimental design and results of Box–Behnken design.

Run	TMP (Bar)	Temperature (°C)	Feed Flow Rate (L/min)	Permeate Flux (L/m^2^h)	Glucose (mg/L)	Fructose (mg/L)	Malvidin-3-O-glucoside (mg/L)
	X_1_	X_2_	X_3_	Y_1_	Y_2_	Y_3_	Y_4_
1	1 (−1)	25 (0)	3.19 (-1)	17.28	300.38	263.3	42.16
2	1 (−1)	20 (−1)	4.93 (0)	38.52	305.09	287.06	43.48
3	2.25 (0)	20 (−1)	6.67 (1)	51.48	296.55	261.87	41.4
4	1 (−1)	25 (0)	6.67 (1)	75.11	307.12	244.79	44.21
5	2.25 (0)	30 (1)	6.67 (1)	56.52	300.74	262.37	43.32
6	3.5 (1)	25 (0)	3.19 (−1)	5.21	316.24	247.625	43.41
7	3.5 (1)	25 (0)	6.67 (1)	34.20	300.9	263.27	43.31
8	3.5 (1)	20 (−1)	4.93 (0)	9.36	312.26	281.35	43.25
9	2.25 (0)	20 (−1)	3.19 (−1)	9.50	299.83	259.39	42.21
10	2.25 (0)	30 (1)	3.19 (−1)	22.68	296.13	260	42.45
11	1 (−1)	30 (1)	4.93 (0)	62.28	304.07	280.03	42.9
12	3.5 (1)	30 (1)	4.93 (0)	14.76	316.47	296.26	41.59
13	2.25 (0)	25 (0)	4.93 (0)	19.44	294.91	314.46	42.23
14	2.25 (0)	25 (0)	4.93 (0)	21.24	285.94	319.97	41.28
15	2.25 (0)	25 (0)	4.93 (0)	27.00	284.62	323.69	43.09

**Table 3:** Optimization results for the clarification of grape marc extract by MF.

Optimized coded level of variables	X_1_: Transmembrane pressure (bar)	1.00
	X_2_: Temperature (°C)	29.01
	X_3_: Feed flow rate (L/min)	5.64
Predicted responses	Permeate flux (L/m^2^h)	65.78
	Concentration of glucose in the permeate stream (mg/L)	305.89
	Concentration of fructose in the permeate stream (mg/L)	274.85
	Concentration of malvidin-3-O-glucoside in the permeate stream (mg/L)	43.73
Overall desirability		0.66

## Data Availability

Data are contained within the article.
